# The *BDNF* Val66Met Polymorphism Modulates Resilience of Neurological Functioning to Brain Ageing and Dementia: A Narrative Review

**DOI:** 10.3390/brainsci10040195

**Published:** 2020-03-25

**Authors:** Donnamay T. Brown, James C. Vickers, Kimberley E. Stuart, Katerina Cechova, David D. Ward

**Affiliations:** 1 Wicking Dementia Research and Education Centre, College of Health and Medicine, University of Tasmania, Hobart 7001, Tasmania, Australia; donnamay.brown@utas.edu.au (D.T.B.); james.vickers@utas.edu.au (J.C.V.); kimberley.stuart@utas.edu.au (K.E.S.); 2 Memory Clinic, Department of Neurology, 2nd Faculty of Medicine, Charles University and Motol University Hospital, 15006 Prague, Czech Republic; katerina.cechova@lfmotol.cuni.cz; 3 International Clinical Research Center, St. Anne’s University Hospital Brno, 65691 Brno, Czech Republic; 4 Division of Geriatric Medicine, Department of Medicine, Dalhousie University, Halifax, NS B3H 2E1, Canada; 5 Centre for Health Care of the Elderly, QEII Health Sciences Centre, Nova Scotia Health Authority, Halifax, NS B3H 2E1, Canada

**Keywords:** ageing, dementia, Alzheimer’s disease, BDNF, brain-derived neurotrophic factor, BDNF Val66Met, cognitive function

## Abstract

Brain-derived neurotropic factor (BDNF) is an abundant and multi-function neurotrophin in the brain. It is released following neuronal activity and is believed to be particularly important in strengthening neural networks. A common variation in the *BDNF* gene, a valine to methionine substitution at codon 66 (Val66Met), has been linked to differential expression of BDNF associated with experience-dependent plasticity. The Met allele has been associated with reduced production of BDNF following neuronal stimulation, which suggests a potential role of this variation with respect to how the nervous system may respond to challenges, such as brain ageing and related neurodegenerative conditions (e.g., dementia and Alzheimer’s disease). The current review examines the potential of the *BDNF* Val66Met variation to modulate an individual’s susceptibility and trajectory through cognitive changes associated with ageing and dementia. On balance, research to date indicates that the *BDNF* Met allele at this codon is potentially associated with a detrimental influence on the level of cognitive functioning in older adults and may also impart increased risk of progression to dementia. Furthermore, recent studies also show that this genetic variation may modulate an individual’s response to interventions targeted at building cognitive resilience to conditions that cause dementia.

## 1. Introduction

Neurotrophins are critical for cellular development, connectivity, plasticity and maintenance in the brain. Brain-derived neurotrophic factor (BDNF), the most abundant neurotrophin, has received significant attention due to a variety of important roles in axonal and dendritic growth and guidance, synaptogenesis, as well as experience-dependent plasticity in adult animal models [1,2,3]. BDNF has been observed secreting from dendrite to axon, from axon to dendrite, in autocrine loops, in paracrine interactions with neighbouring cells and across long distances through neural circuits [2,4]. BDNF may also have a role in the refinement of active neural pathways through activity-dependent strengthening of co-active synapse terminals and elimination of inactive terminals [3]. Temporally, BDNF works within seconds of release to influence synaptic function, over minutes to modify synaptic structure, and over hours to days to change genetic expression and protein synthesis [3]. In this regard, BDNF has a broad influence on both functional and structural forms of neural plasticity throughout the life course (for review, see [5]).

The ‘mature’ BDNF protein is derived from a larger pro-BDNF form and it is yet uncertain whether both the mature and the immature forms of this protein are secreted as a consequence of neuronal activity [6]. Recently, it has been shown that BDNF maturation is a key process for synaptic plasticity. BDNF binds to TrkB receptor and via this pathway promotes dendritic growth, spine density and long-term potentiation [5]. Activation of the pro-BDNF p75^NTR^ receptor reduces neuroplasticity and facilitates long-term depression [7,8]. In this regard, BDNF appears to have a strong mediating role in both long-term potentiation and long-term depression, as well as in influencing broader morphological changes in neurons, likely by signalling changes in actin to effect remodelling [5]. BDNF-immunolabelled cells are present in all cortical regions in both neurons and glia, with a relatively higher density in the insular and temporal cortices, including the hippocampus, basal ganglia and amygdala, in addition to certain frontal regions, and have been observed in white matter adjacent to the cortex [2,9].

The gene for BDNF is located on chromosome 11p14.1 and demonstrates a variety of natural variations in the human population. Of particular focus for this review is the single-nucleotide polymorphism (SNP) at nucleotide 196 in exon 5 of the human *BDNF* gene, involving a guanine to adenine variation that results in the substitution of valine (Val) to methionine (Met). This SNP (rs6265), widely known as the *BDNF* Val66Met polymorphism, has received substantial attention, as hippocampal neurons transfected with the Met version of the SNP express 30% less secretion of BDNF protein upon stimulation than neurons transfected with the Val version, with no differences in constitutive release between these variants [10]. Early reports indicated that *BDNF* Met was associated with reduced localization with synaptic markers and reduced trafficking in transfected cells, lower levels of the metabolic marker n-acetyl aspartate in the human hippocampus, and abnormal medial temporal lobe activation in the N-back working memory task [10,11]. A range of functional deficits and increased anxiety behaviours have been demonstrated in transgenic animal models where the Met version of the *BDNF* gene is expressed [12,13,14]. Studies have demonstrated that mice transfected with two *BDNF* Met alleles show reduced N-methyl-D-aspartate (NMDA) receptor-mediated long-term potentiation and long-term depression in hippocampal and infralimbic medial prefrontal cortex synapses [14,15]. The function of the NMDA receptor has been associated with memory and cognition, particularly within the ageing brain [16,17]. 

Overall, it is assumed that the *BDNF* Val66Met polymorphism leads to a deficit in normal *BDNF* expression and impaired synaptic plasticity through multiple pathways, particularly in the context of activity-dependent functions. Given the critical role of BDNF in neural processes, *BDNF* Val66Met may affect brain and cognitive functioning in the context of ageing and Alzheimer’s disease. Furthermore, given its ties with activity-dependent secretion of BDNF, it is possible that physical activity and cognitive activity may be important modulators to consider. The aim of this review was to examine the potential of the *BDNF* Val66Met polymorphism to alter an individual’s susceptibility to brain ageing and dementia and to further identify pathways through which these effects may be conferred.

## 2. Methods

Articles published up until January 2020 were considered for inclusion. Literature searches were conducted using the PubMed and Google Scholar databases. Key search queries included combinations of the following terms: ‘BDNF’, ‘Val66Met’, ‘A196G’, ‘cognition’, ‘cognitive function’, ‘cognitive functioning’, ‘cognitive decline’, ‘Alzheimer’s disease’, ‘dementia’, ‘cognitive reserve’, ‘physical activity’, ‘exercise’, ‘brain structure’, ‘brain volume’, ‘brain function’, ‘APOE’, and ‘apolipoprotein E’. In addition to these key words, additional search terms of possible relevance were continuously identified from articles identified through database searches. We included articles published in English that focused on both in vivo and in vitro studies of humans and animals. Reference lists of identified articles were also searched to aid in the identification of additional articles of high relevance to the topic.

## 3. Influence of the *BDNF* Val66Met Polymorphism on Brain Structure and Function

Reduced [18] and less efficient [10] hippocampal activation has been reported during memory tasks in healthy young adults who carry *BDNF* Met. Later studies of healthy older adults identified that the presence of *BDNF* Met was associated with a significant reduction in medial temporal lobe activation during the encoding phase of a memory task [19]. Relative to the Val allele, the Met allele has also been linked with reduced neuronal activity in the medial prefrontal cortex in both rodent and human studies [14,20], as well as increased amygdala activation in response to emotional stimuli in human fMRI investigations [21].

In humans, possession of the Met allele has been associated with reduced grey matter in areas of the mid-frontal regions of the brain, fusiform gyrus, amygdala and thalamus [22,23]. In addition, meta-analyses have linked the Met allele to reduced grey matter volume of the hippocampus [24,25]. However, a more recent and larger meta-analysis did not support a link between the polymorphism and hippocampal volume in healthy individuals, indicating that previous associations may have been limited by imaging volume technique and sample size [26]. Ultimately, any association of *BDNF* Val66Met variants with grey matter volume may be, in part, due to developmental effects exerted by the polymorphism, with a recent longitudinal investigation identifying subtle and differential effects of the Met and Val variants on the maturation of grey matter volume in children and young adults [27].

Mixed findings have been reported regarding the relationship between *BDNF* Val66Met and white matter volume and activity, with most studies limited by small to moderate sample sizes. One of the larger studies of 455 adult twins and non-twin siblings reported that the Val allele was associated with a reduction in microstructural integrity of major fibre tracks in various frontal, midbrain and occipital regions, measured by fractional anisotropy [28]. This finding was replicated and further developed in a study that also included radial diffusivity: lower diffusivity was linked to reduced myelination, which was observed in healthy, young Val homozygotes [29]. Notably, these authors suggested that reduced fractional anisotropy and diffusivity can be linked to reduced activity, as well as increased efficiency of these brain regions, thus implying the results may not actually be of a detrimental effect for Val homozygotes. Associations of *BDNF* Val66Met and markers of white matter health or development are, however, inconsistent, with another study finding no relationship between *BDNF* Val66Met and white matter volume or integrity in a sample of 99 young adults [30]. In addition, one study used graph theory to investigate the effect of Val66Met on white matter structural networks in 73 adults (aged in their 30s and 40s) [31]. They found no differences with respect to global efficiency, local efficiency and modularity between Val and Met allele groups. Furthermore, responses to random failures were equal in both genotype groups. However, when targeted attacks to central nodes were modelled, Met carriers were significantly more vulnerable, resulting in reduced connectivity of the white matter structural network [31].

## 4. *BDNF* Val66Met and Cognitive Function

A diverse and not always consistent range of findings regarding the impact of *BDNF* Val66Met on cognitive domains has been reported. A number of studies have reported that healthy, young carriers and homozygotes of *BDNF* Met demonstrate poorer declarative memory, specifically episodic memory [10,18,32,33]. However, statistically significant findings in this domain are not consistently reported [34,35], and it appears that more robust relationships between the polymorphism and cognitive function have been found in samples containing cognitively vulnerable groups, such as older individuals (e.g., [36]) or individuals with schizophrenia (e.g., [10,32]). A recent meta-analysis of almost 6000 participants, comprising a mixed cohort of healthy, psychiatric, younger and older participants, confirmed that Met carriers performed more poorly on declarative memory tasks relative to Val homozygotes [25]. However, when including only the healthy cohorts, this effect became a non-significant trend.

The *BDNF* Met allele has also been implicated in cognitive deficits outside of memory, including reduced visuospatial function performance in individuals with schizophrenia [32,37] and executive function performance in older adults [38]. However, results regarding the relationship between *BDNF* Val66Met and these cognitive functions are also not consistent. For example, a large meta-analysis of 7000 heterogeneous participants reported no link between the *BDNF* Val66Met polymorphism and cognitive functions such as executive functioning, cognitive fluency and general intellectual functioning [39]. Conversely, a study using an auditory distraction paradigm—different, methodologically, from standard cognitive testing—has shown that *BDNF* Val homozygosity may be disadvantageous when inhibitory mechanisms are required, indicating that increased activation associated with the Val allele may not be advantageous for inhibitory effects processed in frontal pathways [40]. A recent systematic review on the topic has confirmed that *BDNF* Val66Met likely has some impact on cognitive function, although the specific direction is difficult to ascertain [41]. Additionally, Toh and colleagues [41] suggest that Met may be advantageous for executive functioning, although broader results are not consistent.

The potential influence of the *BDNF* Val66Met polymorphism on cognition may be age-specific. For example, the determinantal influence of *BDNF* Met on episodic memory, as well as perceptual speed and executive functioning, is typically larger at older ages [42]. In a study of 116 healthy adults aged from 20 to 93 years, the negative association of age and test performance in item and prospective memory was stronger in *BDNF* Met carriers than in Val homozygotes [36]. Other areas of cognitive function may also be exacerbated in older Met carriers. A longitudinal study involving older individuals on perceptual speed tasks over a span of 13 years [43] identified that carriage of the *BDNF* Met allele was associated with a steeper age-related decline in this cognitive ability. Relatively decreased perceptual speed has also been reported in older Met-carrying women [44]. The Met allele has also been implicated in poorer processing speed, delayed recall and general intelligence among elderly individuals [45].

Physiological evidence indicates that older individuals with a *BDNF* Met allele experience decreased hippocampal activation when completing memory tasks, particularly during the encoding process, relative to that of Val homozygotes [19,46]. Changes in brain white matter, specifically the posterior of the corpus callosum, also feature a significant interaction effect of age and the *BDNF* Val66Met polymorphism, with older Met carriers possessing a less dense structure [47], although this is not consistently reported [19]. It has been suggested that BDNF expression decreases with age, which may partly account for older-age cognitive and neurological phenotypes [48]. This ageing-related decline in BDNF availability may be further exacerbated by the Met allele, which is associated with less BDNF activity-dependent secretion [10]. However, null and inverse findings regarding the cognitive implications of *BDNF* Val66Met variants have also been reported. For example, an examination of *BDNF* Val66Met and performance across multiple cognitive domains in a sample of older Chinese men found no difference between Val/Val, Val/Met or Met/Met groups [49]. This study may, however, have been underpowered to detect the small effects that have been reported elsewhere (*n* = 161). In a longitudinal study of older adults, Met carriers performed more poorly on a task of executive function at study entry but did not exhibit a decline in performance, which was present in Val homozygotes, over the 10-year follow-up period [50]. Enhanced executive function in Met carriers, particularly in inhibitory tasks, has also been reported in other studies [51,52]. One study found this positive Met effect for executive functioning in a cohort with an average age of 79 but not in their second cohort with an average age 64 [53]. Consistent with what is found in young adults, *BDNF* Val or Met alleles’ positive or negative effects on cognitive performance in older subjects may depend on the specific task requirements and goals.

Taken together, the current state of evidence indicates that in older adults without clinically significant cognitive impairment the Met allele may be somewhat detrimental to certain cognitive functions and not associated with performance in others.

## 5. Exploring the Link Between the *BDNF* Val66Met Polymorphism and Dementia Due to Alzheimer’s Disease

The rapidly increasing number of people with dementia throughout the world presents an immense health, social and economic challenge, for which we currently have no evidence-based capacity to prevent, halt or cure. Many of the neurodegenerative diseases that cause dementia, such as Alzheimer’s disease (AD), are characterised by the development of specific pathological features and the progressive loss of subsets of neurons and their connections [54]. Given the significant role that BDNF exhibits in the support and survival of specific neuronal populations, including those affected in AD, BDNF may too play an important role in the pathophysiology of AD.

BDNF and its receptor TrKB are both reduced in expression in the AD brain [55], with BDNF levels being most significantly reduced in the hippocampus and parietal cortex [56]. Levels of circulating BDNF protein can be established from blood samples and have been reported to be reduced in psychiatric conditions [57] as well as in individuals with mild cognitive impairment (MCI; [58]), the latter proposed to be a potential precursor state for AD. However, varied reports of increased [59], decreased [60] and unchanged [61] BDNF levels in neurodegenerative conditions such as AD have been found. Increased BDNF in serum has been linked with specific memory deficits in AD [62], and decreased BDNF has been associated with the presence of *APOE* ε4 and apathy in subsets of AD [63]. In a prospective study of 2131 older individuals without dementia, higher serum BDNF level, but not the *BDNF* Val66Met genotype, was associated with a reduced risk of subsequently developing dementia [64]. Interestingly, subsequent subgroup analysis in this investigation showed that the association of high BDNF levels and lower risk of dementia was found solely in women with a college degree. However, others have found the *BDNF* genotype does play a role in the cognitive performance of individuals at higher risk of developing dementia. High amyloid-beta load in the brains of cognitively normal older *BDNF* Met carriers, relative to Val homozygotes, has been linked with worse episodic memory and executive function [65] and with a steeper trajectory of cognitive decline over time [66]. In a similar study of older people without dementia, carriage of the Met allele was associated with greater entorhinal cortex atrophy and lower Mini-Mental State Examination scores specifically in those individuals with amyloid positivity only [67].

Relatedly, greater hippocampal atrophy over a three-year period has been reported in Met carriers who had higher levels of AD-related pathology [65], and, in autosomal-dominant AD, Met carriers have significantly worse memory performance, lower hippocampal metabolism and increased levels of pathological tau [68]. Such findings may explain why inheritance of a Met allele is associated with a higher risk of conversion from MCI to AD [69]. In cohorts including subjective memory decline, MCI and dementia, the detrimental effects of amyloid positivity (for example, from fluid biomarkers or PET imaging) on brain connectivity (hippocampus to medial/frontal connectivity) were higher in *BDNF* Met carriers [70]. Finally, in older adults possessing gene mutations linked to dominantly inherited AD but without dementia, *BDNF* Met carriage was associated with more substantial memory decline and hippocampal volume loss, as well as increased cerebrospinal fluid (CSF) tau levels, relative to Val homozygotes [71].

It is, however, important to acknowledge that contradictory findings have also been reported with regard to the associations of *BDNF* Val66Met and risk for dementia. For instance, in a Japanese sample of 487 participants with AD and 471 matched controls, *BDNF* Val homozygosity was observed more commonly among the participants with AD [72]. Similar results with regard to a heightened AD risk incurred from *BDNF* Val have been reported from within an Italian sample of Caucasians [73]. Specifically, a *BDNF* Val allele was more frequently observed within AD patients than among healthy volunteers. Such results are further consistent with those of a Hungarian sample, in which *BDNF* Val homozygosity was present in 59% of AD cases but only 32% of healthy controls [74]. Some degree of the inconsistencies in reported relationships between *BDNF* Val66Met and AD are likely due to differences in the diagnostic criteria used for AD and differences in population distributions of *BDNF* Val66Met alleles.

Thus, although the relationship between serum BDNF and AD symptomology may be unclear, current research suggests that inheritance of *BDNF* Met leads to more severe or more rapidly progressing cognitive deficits in the presence of AD pathology, in most cases. However, the presence of a *BDNF* Met allele does not appear to directly and independently affect an individual’s risk for dementia. These studies raise the prospect that many effects of the *BDNF* polymorphism in ‘healthy’ older cohorts, as described above, may have been influenced by the presence of sub-clinical AD and other neurodegenerative pathology.

## 6. Interactions between the *APOE* Gene and the *BDNF* Val66Met Polymorphism

Age and family history are the major risk factors for dementia, with risk of AD rising exponentially after the age of 65 years. The most significant non-mutation genetic risk factor for AD is a common variant in the *APOE* gene. Specifically, the presence of one or more *APOE* ε4 alleles increases the risk of AD in older adults in a dose-related fashion [75,76]. With regard to brain structure and function, a growing body of evidence reports interactions between *APOE* and *BDNF* Val66Met variants.

In one study of healthy older adults, an interaction effect of *BDNF* Val66Met and *APOE* was identified, in which the presence of both of the compromising alleles (Met and ε4) was linked to significantly reduced hippocampal activation during memory tasks [77]. Other imaging studies have found that carriers of both *BDNF* Met and *APOE* ε4 possess a lack of neural compensatory capacity when challenged by AD [78]. Specifically, during ageing, the thickness of the precuneus and posterior cingulate cortex decreases more markedly in *BDNF* Met carriers, and the *BDNF* Met/*APOE* ε4 combination is associated with steeper entorhinal cortex atrophy in MCI/AD cases, as well as steeper performance decline on memory tests [78]. The combination of amyloid-beta deposits in the brain with both *APOE* ε4 and *BDNF* Met has been related to the steepest declines in memory and language cognitive domains in older individuals without dementia [79]. This latter study estimated *BDNF* Met carriers with the *APOE* ε4 allele would have clinically significant cognitive impairment in 3 years, as compared to 10 years if the individual was an *APOE* ε4 carrier and *BDNF* Val homozygote. Furthermore, a protective effect of *BDNF* Val on episodic memory performance in older adults without dementia has been reported [80]. In this study of 433 healthy older adults, no difference in performance was observed between *APOE* allele types in *BDNF* Val homozygotes, but the protective effects of *APOE* ε2 and the detrimental effects of *APOE* ε4 were observed in *BDNF* Met carriers. Consistent with other findings [19,79,80], a recent study on amnestic MCI has likewise indicated that inheritance of the *APOE* ε4/*BDNF* Met combination was associated with the worst memory performance when compared to other polymorphic combinations [81].

In combination, these studies have demonstrated that an interaction between *APOE* and *BDNF* Val66Met variants likely explains more variance in brain and cognitive outcomes than each gene variant explains individually. At the very least, an additive detrimental effect of both of these at-risk allele types is exerted on memory performance, its associated brain regions and patterns of brain connectivity [82].

## 7. Physical Activity, *BDNF*, and Risk of Cognitive Decline

It has been suggested that approximately a third [83,84] to half [85] of dementia cases may be effectively preventable by addressing major modifiable factors, such as depression, mid-life hypertension, smoking, mid-life obesity, diabetes, physical inactivity and low educational attainment. Low physical activity in particular has been identified as a major population-attributable risk factor for dementia [83,84,85], as well as increasing the risk of other lifestyle-related diseases associated with increased susceptibility to dementia, including obesity, type 2 diabetes and cardiovascular diseases [86]. Consequently, there is substantial interest in whether increasing physical activity and exercise may constitute an intervention to reduce risk or progression of dementia. Meta-analyses of observational data support a role for exercise in improving cognitive performance, although the results of relevant prospective trials are mixed [87].

In animal studies, brain BDNF is increased in the hippocampus and cerebral cortex following exercise [88], as well as following environmental enrichment which also promotes physical activity (e.g., [89,90,91]). Blocking BDNF action inhibits the exercise-mediated increase in synaptic proteins [92]. Furthermore, exercise can counteract the detrimental effects of other variables that may cause a reduction in BDNF levels, such as stress [93]. In humans, peripheral BDNF is increased with a single session of exercise, with intensified session-based effects and increased resting BDNF with additional subsequent sessions [94]. However, this meta-analysis also reported that these benefits may be modulated by sex, with women experiencing a decreased effect [94]. The beneficial effects of exercise on BDNF secretion also appear to attenuate with age in rodents, with this effect reduced in older mice but still present [95], although this is yet to be clarified in human research.

Still, multiple trials have demonstrated improvements in cognitive functioning and increased levels of serum BDNF following exercise interventions. For instance, in participants with amnestic MCI, acute aerobic exercise and acute resistance training led to improved cognitive functioning compared to control in two separate trials [96,97]. In these studies, increased serum BDNF levels were additionally observed among the participants who completed the acute aerobic exercise program. In a 16-week multimodal physical exercise program involving older cognitively healthy individuals and individuals with MCI, physical training reduced blood-based pro-inflammatory markers, increased BDNF plasma levels and improved cognitive functioning among those with MCI [98]. While upregulation of *BDNF* expression is presumed to account for some of the exercise-related cognitive benefits, it is interesting to note that the cognitive improvements observed after aerobic exercise in the study by Tsai and colleagues [96] were not significantly correlated with the observed increases in serum BDNF.

With respect to the *BDNF* Val66Met polymorphism, a longitudinal investigation involving over 700 community-based older adults demonstrated that lower baseline physical activity was linked to increased incidence of cognitive difficulties and dementia, a risk that increased incrementally with the number of Met alleles present [99]. Additionally, a longitudinal analysis following older adults over a 12-year period identified a possible sex interaction, in which physical activity was positively associated with cognitive function in male *BDNF* Val homozygotes but not in male Met carriers [100]. In contrast, physical activity was not associated with cognition in women of either genotype. A similar finding, that physical activity was associated with improved executive functioning and a slower decline in this cognitive domain over a 9-year period solely in older Val homozygotes, has also been reported [101]. In this study, however, sex was not a moderating factor on the relationships between *BDNF* Val66Met, exercise and cognitive performance. In a separate cross-sectional study, older Val homozygotes who exercised more regularly had, on average, greater volume in hippocampal and temporal regions when compared to Met carriers who exercised a similar amount [102].

Exercise has been used as an intervention to improve cognitive function in older adults, but the potential interaction with *BDNF* Val66Met status remains unclear. For example, one study determined that improvements in episodic memory performance following exercise were dependent on Val homozygosity [103], but Nascimento and colleagues [104] reported that *BDNF* Val66Met was unrelated to any cognitive outcome in their study. In a younger-to-middle-aged (30–55 years old) sample, exercise appeared to compensate for lower performance on cognitive measures of working memory in *BDNF* Met carriers relative to Val homozygotes [105]. In those even younger (21–35 years), no differential effect of exercise on performance in locomotor learning was linked to variation in *BDNF* Val66Met [106].

Although difficulties in comparing available studies on this topic are evident due to methodological differences in both exercise intervention and assessment of cognitive function, these findings indicate that age may also play a role in the interaction between *BDNF* Val66Met and exercise. This is in line with the resource modulation hypothesis, in that gene-related effects on cognitive function are more evident in older participants [42,100,107]. While there may be no overt effect of exercise in younger adults, middle-aged *BDNF* Met carriers who exercise and generally engage in more physical activity may be able to avoid a negative Met-specific cognitive phenotype, while older adults, particularly those with MCI or cognitive difficulties, may experience greater cognitive benefits from exercise-induced secretion of BDNF if they are Val homozygotes. Additional cross-sectional and longitudinal research covering a wide range of age groups measuring cognition in a consistent manner would be of further benefit to examine this hypothesis.

## 8. *BDNF* and Cognitive Reserve

Similar to physical activity, cognitive activity, often reflected by education level, has been identified as one of the major modifiable risk factors for dementia. Although a higher level of education may exert some of these protective effects through its association with socioeconomic status and greater access to healthcare, engaging in further education may also directly benefit the development, structure and function of the brain [108,109].

The cognitive reserve hypothesis posits that greater engagement in cognitively stimulating activities across the lifespan results in increased protective neural compensation and heightened neural efficiency, which results in a later onset of dementia despite having little effect on disease pathology onset or rate of accumulation [110,111]. Stern [110] suggests that individuals with higher cognitive reserve have, on average, higher levels of neuropathology prior to experiencing behavioural changes, displaying resilience to the effects of the pathology. This has subsequently been supported by evidence from a range of studies, in which greater brain connectivity and maintenance of cognitive function have been directly associated with cognitive reserve variables in the presence of MCI and AD [112,113]. The effects of cognitive reserve are not restricted to pathological functioning, and the level of cognitive reserve correlates positively with performance in a range of cognitive domains in healthy older adults [114]. This cross-sectional investigation also reported that a positive association of cognitive reserve and executive functioning was present in *BDNF* Val homozygotes but not in Met carriers. In a subsequent 36-month longitudinal investigation of this same cohort, cognitive reserve was associated with the rate of change in executive functioning only when the interaction with *BDNF* Val66Met was considered, with the low-cognitive-reserve/*BDNF* Met carrier participants the sole group to show the beginnings of cognitive decline [115].

Similar to physical activity, emerging evidence also points to a role of cognitive activity in stimulating the release of BDNF protein [116]. In this small-scale intervention study of older women with MCI (*N* = 44), the authors observed a parallel increase in both serum BDNF level and cognitive performance following 24 sessions of computerised mental training. The role of the *BDNF* polymorphism was analysed in a similar study assessing the cognitive outcomes of a computerised brain-training program in 27 older adults who had experienced heart failure [117]. In this investigation, although serum BDNF levels increased following the intervention, no differences in BDNF levels or cognitive performance were observed due to differing *BDNF* Val66Met variants. Nonetheless, with a small effect size expected with regard to the modulatory effects of *BDNF* Val66Met on cognitive and serum outcomes following cognitive stimulation, these investigations are likely underpowered to find support, or otherwise, for such a hypothesis.

Relatedly, this *BDNF* genotype may also impact interventions designed to improve or stabilise cognitive function and cognitive reserve, potentially acting as a bulwark against neurodegenerative conditions such as dementia. The Tasmanian Health Brain Project is a long-term pragmatic interventional study that is investigating the potential cognitive benefits for older adults (50–79 years of age) re-engaging in formal education. This study has found that across all older adults, the cognitive domain of language processing benefits most from engagement in university education [118]. Furthermore, the *BDNF* Val66Met polymorphism may influence who may benefit from such interventions, with Val homozygotes deriving less benefit than Met carriers [119]. Specifically, Met carriers who were not engaged in education showed a decline in language processing function over time, whereas Met carriers in the intervention group gained a benefit in this cognitive domain in a dose-related fashion relative to the amount of study that they had undertaken.

## 9. Summary and Limitations

The BDNF protein plays an important role in mediating the interaction between the external environment and internal neurological function and capacity. Met allele carriers for the *BDNF* Val66Met polymorphism may represent a ‘vulnerable’ group in the face of a brain challenge such as a major neurological disease like AD. From animal studies and investigations of human brain plasticity, the Met allele version appears to be associated with a somewhat deficient plasticity response to experience. However, some of these effects may be direct or indirect and potentially too subtle to be meaningful for future translational studies. In our studies, the *BDNF* Val66Met polymorphism may be influencing how an individual is accessing the benefits of a lifetime of exposure to cognitively stimulating experiences to build cognitive reserve and other areas of cognitive capacity that may help reduce susceptibility to dementia [114,115,119]. In this regard, it may be vital that people with the vulnerable genotype are engaged in mentally stimulating activities serving as a bulwark against dementia. Specific genetic combinations, such as that of *APOE* and *BDNF* Val66Met, may further identify cognitively vulnerable people who may be particularly advised to consider how they can reduce their risk of dementia outside of the influence of ageing and genetic inheritance. Although not reported here, the *BDNF* Val66Met polymorphism has also been linked to a deleterious response to stress and trauma [120], which may play a role in cognition and subsequently dementia, given the relation between neurodegenerative changes and an abnormal stress response in AD [121].

Although much of the research has focused on *BDNF* Val66Met allele variation, Chen and colleagues [12] note that it is possible that some of these effects could be related to another component of the *BDNF* gene or that *BDNF* Val66Met is mediating a downstream effect or the effect of another gene. Many polymorphisms in the *BDNF* gene have been identified, including rs11030104, rs16917204, rs7103411, rs6265 and rs2030324, which may have an interaction effect or better explain some of the variations in the levels of BDNF in certain contexts [122]. A further limitation is that most human-based studies on *BDNF* Val66Met often, but not always, exclusively consider either Caucasian or Asian samples, with participants excluded from analyses if not part of the majority. This is because there is potential for different patterns associated with Asian and European populations, which may be related to a unique location of the SNP in each ethnic group [123].

## 10. Conclusions

On balance, research to date indicates that the *BDNF* Met allele is potentially associated with a detrimental influence on the level of cognitive functioning in older adults and may also impart increased risk of progression to dementia through multiple pathways.
